# Framework and Practical Guidance for the Ethical Use of Electronic Methods for Communication With Participants in Medical Research

**DOI:** 10.2196/33167

**Published:** 2022-04-20

**Authors:** Atsushi Kogetsu, Kazuto Kato

**Affiliations:** 1 Department of Biomedical Ethics and Public Policy Graduate School of Medicine Osaka University Suita, Osaka Japan

**Keywords:** online communication, electronic methods, online recruitment, electronic informed consent, e-IC, digital consent, online consent, data communication, digital health

## Abstract

Online communication with participants, including online recruitment, electronic informed consent, and data communication, is one of the fields to which information and communication technology (ICT) has been applied in medical research. Online communication provides various benefits, especially for genome research and rare disease research. However, ethical challenges that are derived from or exacerbated by online communication need to be addressed. Here, we present an overview of such ethical issues and provide practical guidance for the ethical implementation of ICT. We specify the ethical issues in the context of using online communication for medical research by an analysis based on the eight ethical principles for clinical research. Informed by this ethical context, we then develop a novel framework for the governance of medical research involving ICT, which consists of eight categories: five research processes (ie, design of research, recruitment, informed consent, data communication, and dissemination and return of results) and three overarching perspectives related to multiple processes of research (ie, access to research and online dialog, community involvement, and independent review). Finally, we present a practical guidance chart for researchers, patient partners, independent reviewers, and funding agencies. We believe that our study will contribute to the ethical implementation of online communication in medical research.

## Introduction

One of the applications of information and communication technology (ICT) in medical research is a variety of online communications with participants, including recruitment, informed consent, and sharing of research results [1-4]. The use of the internet is expected to enable continuous communication without spatial restrictions [3]. This is especially beneficial in rare disease research because it is difficult to recruit a sufficient number of participants in geographically limited areas. As clarified by the Rare and Undiagnosed Diseases Study (RUDY) in the United Kingdom and Japan, digital platforms enable the effective recruitment of participants [5-7]. Additionally, the RUDY project demonstrated that digital technologies facilitate dialog and collaboration between patients and researchers [5-7]. This type of active patient involvement encouraged by digital tools is referred to as participant-centric initiatives [8,9]. Another research area where ICT use is expected to bring about significant change is human genome research. Since genome cohort studies such as the “All of US” study in the United States aim to recruit a large number of participants, registering and answering an online questionnaire should prove to be efficient and helpful for participants [10]. Moreover, long-term communication with participants is more important for genome research projects because the results of individual analyses can change as data are accumulated over time [11].

At the same time, online communication can raise new ethical challenges such as the digital divide, concerns about invasion of privacy, how to assure understanding of information for consent, and methods for authenticating participants [12-15]. To maximize the benefits of ICT, it is crucial to deal with such ethical challenges. Previous studies focused on individual ethical issues; however, there has been no attempt to provide an overview of the ethical issues that need to be addressed in implementing online communication with participants. Although some countries already have regulations or recommendations regarding electronic methods, such as guidance on the use of electronic consent released by the US Food and Drug Administration [16], their scope is limited. In Japan, the newly revised ethical guidelines for medical research established in 2021 include descriptions about online informed consent, but few details are specified. Therefore, the purpose of this viewpoint is to provide an overview of ethical issues and present a practical guide for implementing online communication in medical research.

We initially performed an analysis based on the eight ethical principles for clinical research proposed by Emanuel et al [17], referred to as the Emanuel Framework (EF). We specified the ethical issues in the context of online communication with participants through each principle of the EF: collaborative partnership, social value, scientific validity, fair participant selection, favorable risk-benefit ratio, independent review, informed consent, and respect for participants. The lead author (AK) carried out a literature search and both authors performed analyses. We then developed a new framework composed of the issues specified by the analysis, after receiving feedback from the researchers of the medical and genomics research projects under the same research program that funded this study, sponsored by the Japan Agency for Medical Research and Development (AMED). Finally, we propose a practical guide for researchers, patient partners, independent reviewers, and funding agencies.

## Ethical Analysis Based on the EF

### Collaborative Partnership

The first key principle in the EF emphasizes that during the course of medical research, creating a good partnership among medical researchers, participants, and other members of the project is crucial. Several key issues emerge when examining situations to conduct medical research using ICT.

First, there is a possibility that research projects have difficulties in finding representatives of participating communities, although ICT enables existing community members to communicate more easily. For example, for large-scale research projects that target multiple countries, it is a challenge to decide who represents the target population. It may be necessary to redefine our understanding of “community” as well as community “representatives.” This newly understood community would include online patient networks that have been attracting attention in recent years [18-21].

Second, a community’s distinct values, circumstances, culture, and social practices should be respected even when employing electronic methods. If most of the community members are not familiar with the internet or are reluctant to send their information online, electronic methods should not be implemented.

Third, digital tools make collaboration more diverse, enabling more casual patient involvement such as feedback online [22]. One challenge is how to share responsibility with such casual involvement. A fair distribution of the tangible and intangible rewards of research among the partners based on each contribution is also a challenge.

### Social Value

When conducting medical research, assessing and enhancing its social value are essential as well as protecting participants. Using ICT can enhance the social value of medical research. Performing large-scale studies with electronic methods leads to an increase in the number of beneficiaries. The value of the research can also be enhanced with the secondary usage of the data online, which may be especially true for studies using genomic data. Research results can be disseminated more widely with the internet [23]. Moreover, the use of multimedia can improve accessibility and understanding.

However, caution is needed to prevent adverse impacts on existing health care infrastructure and its sustainability when communicating directly with participants across countries through online systems. This is especially true when returning individual genetic analysis results because novel therapeutic strategies based on these results may create an additional burden on existing health care systems.

### Scientific Validity

Scientific validity, the third principle of the EF, is one of the fundamental factors for ethical research. When utilizing ICT for medical research, it should be taken into account that the use of ICT can positively influence the scientific validity and reliability of the research design. Regarding study designs, a sufficient sample size is important to ensure scientific validity. For rare disease research, recruiting a sufficient number of participants online makes the studies more scientifically valid.

However, the ease of this approach varies depending on the frequency of ICT use and differing values toward privacy. For instance, online recruitment may be ineffective for people who do not have access to the internet, are unfamiliar with it, or do not want to enter their personal information online. This may cause selection bias [2,19,24-27]. At the time of registration, especially when there is a reward for participation, preventing duplicate registration of participants is also important to ensure scientific validity [28].

### Fair Participant Selection

Research participants must be selected primarily based on scientific objectivity. The online platform allows individualized recruitment, which makes it possible to achieve a fairer selection of participants by ensuring accountability for the selection [29].

For research projects that target vulnerable people, careful consideration is necessary. It is conceivable that the use of electronic methods to facilitate participation in research will enable vulnerable individuals to participate in more research owing to their medical or economic circumstances, which could cause overexploitation or insufficient understanding of the projects. Measures should be taken to protect the potential participants with such vulnerabilities from the risks associated with research participation.

### Favorable Risk-Benefit Ratio

When carrying out research, potential risks and benefits for individual participants should be assessed and explained. Based on this principle, the risks associated with using electronic methods should be identified, described, and minimized. These risks include data leakage during online data transfer and impersonation by others in the digital authentication system for participants [15]. Another risk is miscommunication caused by the lack of nonverbal information when using certain kinds of online tools [30].

In the process of returning individual genetic analysis results, sufficient medical, psychological, and social care for the patients are absolutely required. When returning results through online systems, it should be noted that there may be an increased risk of failing to ensure that such considerations are made.

### Independent Review

Independent review mechanisms are usually determined by laws and regulations, and vary depending on countries or institutions. In traditional research projects, researchers generally recruit participants from their own countries and therefore only need to follow the rules of their countries. However, online recruitment may encourage participation across countries and institutions. Therefore, there is often a lack of laws and regulations concerning the detailed procedures for independent review of research protocols when the research is planned in one country and participants are recruited from other countries, as in so-called direct-to-participant (DTP) recruitment. A detailed analysis of these issues is beyond the scope of this paper; however, some possible solutions have already been proposed [31].

In an independent review, reviewers should be competent; however, it is currently very difficult to adequately assess research using online communications from scientific, ethical, and technical perspectives due to the lack of research or guidance on practices for implementing communications using electronic methods. In particular, questions concerning how a secure research system can be built and what kind of privacy risks associated with online recruitment may occur cannot be fully assessed without a high level of expertise [32]. Therefore, the availability of reports and guidance, as well as expert advice, is crucial.

### Informed Consent

Informed consent requires demonstration of respect for the autonomy of individuals, and online consent is no exception. Online informed consent enables prospective participants to make their participation decisions at their own pace, which is a major advantage in reducing psychological impact [33]. It is also important to ensure individual authentication for voluntary research participation [15].

In terms of recruitment procedures, it is necessary to assess whether online recruitment is culturally, politically, and socially acceptable. For example, due consideration should be given to the recruitment of prospective participants when using online behavior histories or social networks, if these are considered to be potential invasions of privacy [27].

It is important that the information presented online is complete, accurate, and not overwhelming. Adjusting the amount of information presented at one time and using multimedia are expected to improve the understanding of the prospective participants [33,34]. However, it is necessary to provide information tailored to individual literacy rather than providing a uniform explanation. Furthermore, to obtain informed consent with the full understanding of the participants, the opportunity to ask questions must also be guaranteed in online informed consent. Unless face-to-face informed consent is provided, such as through a video conferencing system, a more careful assessment is required as to whether candidates fully understand the information presented [13,33]. In addition, it should be noted whether the means of symbolizing consent, such as electronic signatures and clicks for online informed consent, are sufficiently accepted in the communities to which the candidates belong. Finally, as indicated in the US Guidance, the method of obtaining informed consent from a participant’s legally authorized representative should also be determined in advance [16].

### Respect for Participants

Communication with participants online can help with monitoring health and well-being so as to minimize harm, particularly through subjective assessments. Online methods may help to facilitate communication in terms of providing participants with information about research progress and results, related health care, and treatment. However, when, how, and what information is appropriate to provide may vary from person to person and, as such, these issues should be considered to suit individual participants where possible.

Regarding confidentiality, various security challenges raised by online communications must be addressed [14]. It should be noted that additional data that can be collected, such as location data, may infringe on the privacy of participants [35]. To ensure security, it is necessary to establish a reliable method of authentication [15].

## Overview of the Ethical Issues in Online Communication With Participants

**Figure 1:**
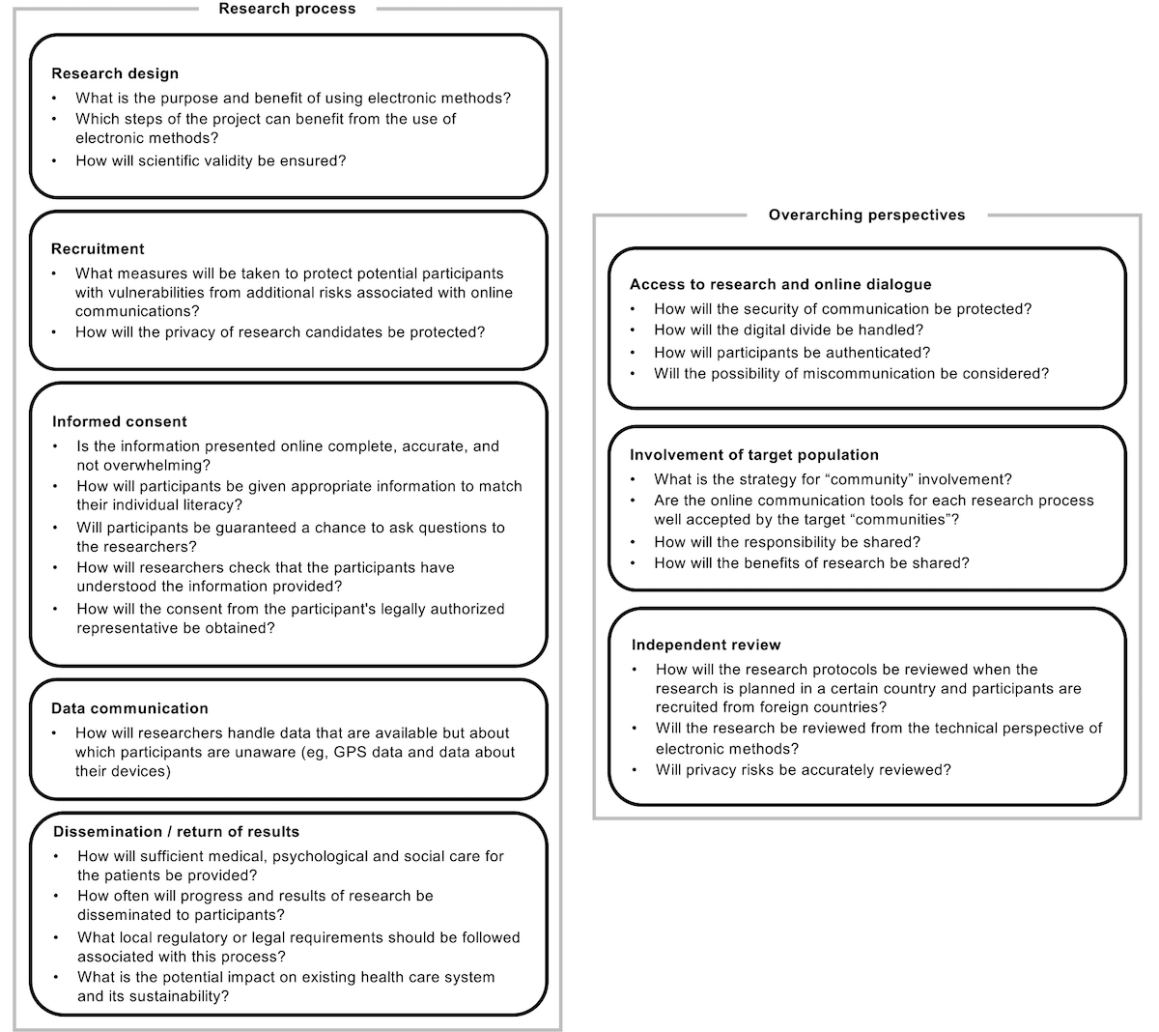
Framework for the governance of genomics research involving information and communication technology. The five boxes on the left show the research process and the three boxes on the right show overarching perspectives related to the five processes. Each box contains the main benchmarks for that issue or process.

Informed by the EF ethical analysis, we have schematically presented the interrelationships among the issues based on the actual processes of medical research and formulated a novel framework for the governance of medical research involving ICT (Figure 1).

The framework is designed for any research involving communication with participants, not for studies that involve digital technology in general (eg, research drawing on electronic medical records without any online communication with participants). The framework covers eight elements. The first five elements (left side of Figure 1) are the processes involved in medical research using ICT, which cover the design of research, recruitment, informed consent, data communication, and dissemination of progress and results, including the return of individual genomic analysis results. The other three elements (right side of Figure 1) are overarching perspectives. They cover access to research, community involvement, and independent review. In each category, benchmarks are described to show what issues are involved.

The “design of research” category includes the purpose and benefit of the use of electronic methods. In addition, it should be taken into account that online communication would affect scientific validity. This is especially true when recruitment and data communication through online systems are employed. The category of “recruitment” includes the issues related to risks when recruiting participants online. Researchers must consider that online recruitment would have additional risks for participants with vulnerabilities and that some of their data may be available before the participants have given consent. The category of “informed consent” includes the benchmarks associated with information presented online, the understanding of participants, and consent from the participant’s legally authorized representative. The category of “data communication” covers the situation in which participants manage their own data or when data are generated by the participants’ own devices. The category of “dissemination and return of results” includes the benchmarks related to not only individual participants but also the target community.

Security issues, digital divide, authentication of participants, and the risk of miscommunication are important factors in considering access to the research system and online dialog between researchers and participants. The “involvement of target population” category includes the benchmarks associated with the strategy and tools for “community” involvement, as well as sharing responsibility and benefits. The category of “independent review” includes the benchmarks regarding the procedures for independent review of DTP recruitment and competency of the reviewers.

## Implications for Practice

**Figure 2:**
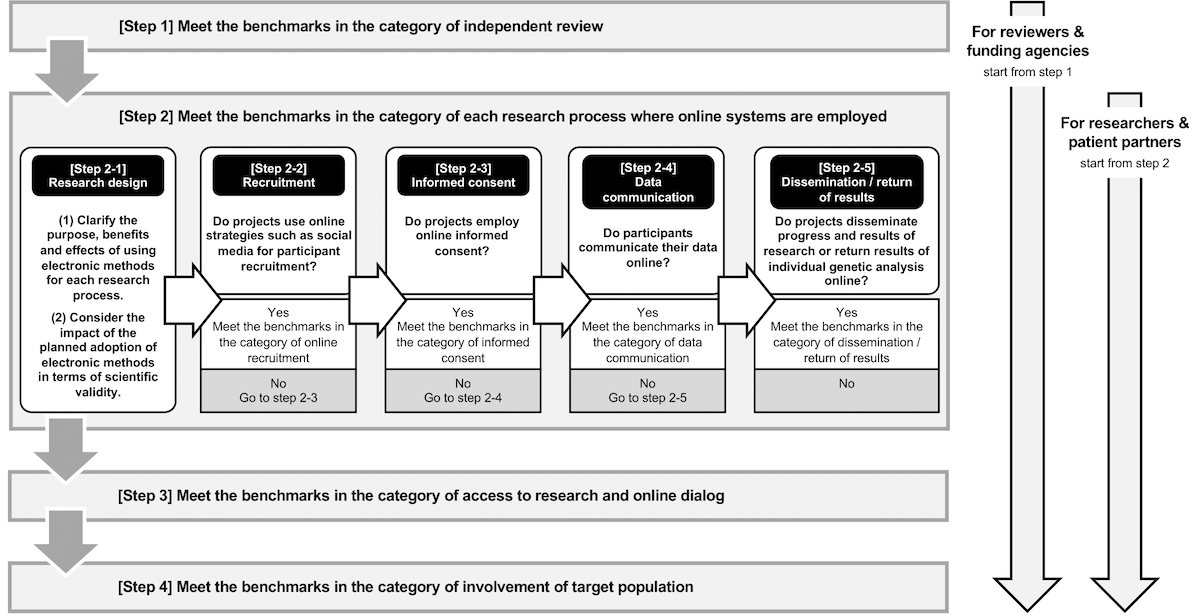
Guidance chart for ethical research using online communication with participants. This guidance chart mainly consists of four steps. Step 1 is for independent reviewers and funding agencies, and steps 2, 3, and 4 are for reviewers, funding agencies, researchers, and patient partners.

Based on this framework, we present a practical guide in the form of a chart for researchers, patient partners, independent reviewers, and funding agencies (Figure 2). This guidance chart mainly consists of four steps: (1) meet the benchmarks in the category of independent review, (2) meet the benchmarks in the category of each research process where online systems are employed, (3) meet the benchmarks in the category of access to research, and (4) meet the benchmarks in the category of patient involvement.

Depending on the users of the chart, the first step is either step 1 or step 2. For independent reviewers and funding agencies, the first step is to ensure that the reviewers are competent to review this type of research using online communications with participants, especially from the perspectives of technology and privacy risks. In cases where they are not competent, they need to ask ICT experts for help and review the literature to address new ethical issues such as the means of symbolizing consent in the process of informed consent.

Steps 2 to 4 are for independent reviewers, funding agencies, researchers, and patient partners. In the second step, researchers should clarify the purpose, benefits, and effects of using electronic methods for each research process, as well as the impact of the planned adoption of electronic methods in terms of scientific validity. Subsequently, researchers and patient partners should address the ethical issues related to each research process where they plan to employ online systems for communication with participants, from recruitment to dissemination of research results. Concrete issues that need to be addressed can be found in the benchmarks in the framework categories shown in Figure 1. Independent reviewers should review each step by carefully examining the content of research protocols that address these ethical aspects.

The third and fourth steps focus on the overarching perspectives that need to be ensured in all of the processes of research projects. Even if a research project plans to employ online communication only for one process such as online informed consent, the ethical issues included in the category of access to research and online dialog, and involvement of the target population should be addressed.

For funding agencies, in addition to making sure that the relevant benchmarks are met, it is desirable to ensure that adequate funding is available for the costs of addressing ethical issues in each step, particularly in steps 1, 3, and 4. Regarding step 1, funding may be required to ask ICT experts for help and access to useful literature. In step 3, large investments are sometimes required to address security issues and to implement appropriate authentication systems.

## Conclusion

As ICT becomes implemented and applied more widely, it is hoped that it will be used actively in medical research. In some countries, electronic methods are increasingly being employed in research projects. Online systems should be utilized carefully while keeping in mind the ethical considerations that have been described in this article. We believe that our ethical framework and the guidance chart can help to make electronic strategies ethically sound and acceptable.

## Abbreviations

AMED: Japan Agency for Medical Research and Development
DTP: direct-to-participant
EF: Emanuel Framework
GRIFIN: The Advanced Genome Research and Bioinformatics Study to facilitate Medical Innovation
ICT: information and communication technology
RUDY: Rare and Undiagnosed Diseases Study
